# Supplementary material for: Leptin Levels of the Perinatal Period Shape Offspring’s Weight Trajectories through the First Year of Age
Source: Nutrients. 2022 Mar 30;14(7):1451. doi: 10.3390/nu14071451 (PMC9003253; doi:10.3390/nu14071451)
Supplement: Supplementary file 1 [file nutrients-14-01451-s001.zip › nutrients-1631017-supplementary.pdf]

Table S1. Weight percentile at 12 month of age in the three different groups of children.

groups	< 10° C	10°-50 ° C	50°- 90° C
FT	2	13	1
PT	3	7	6
IUGR	7	4	2

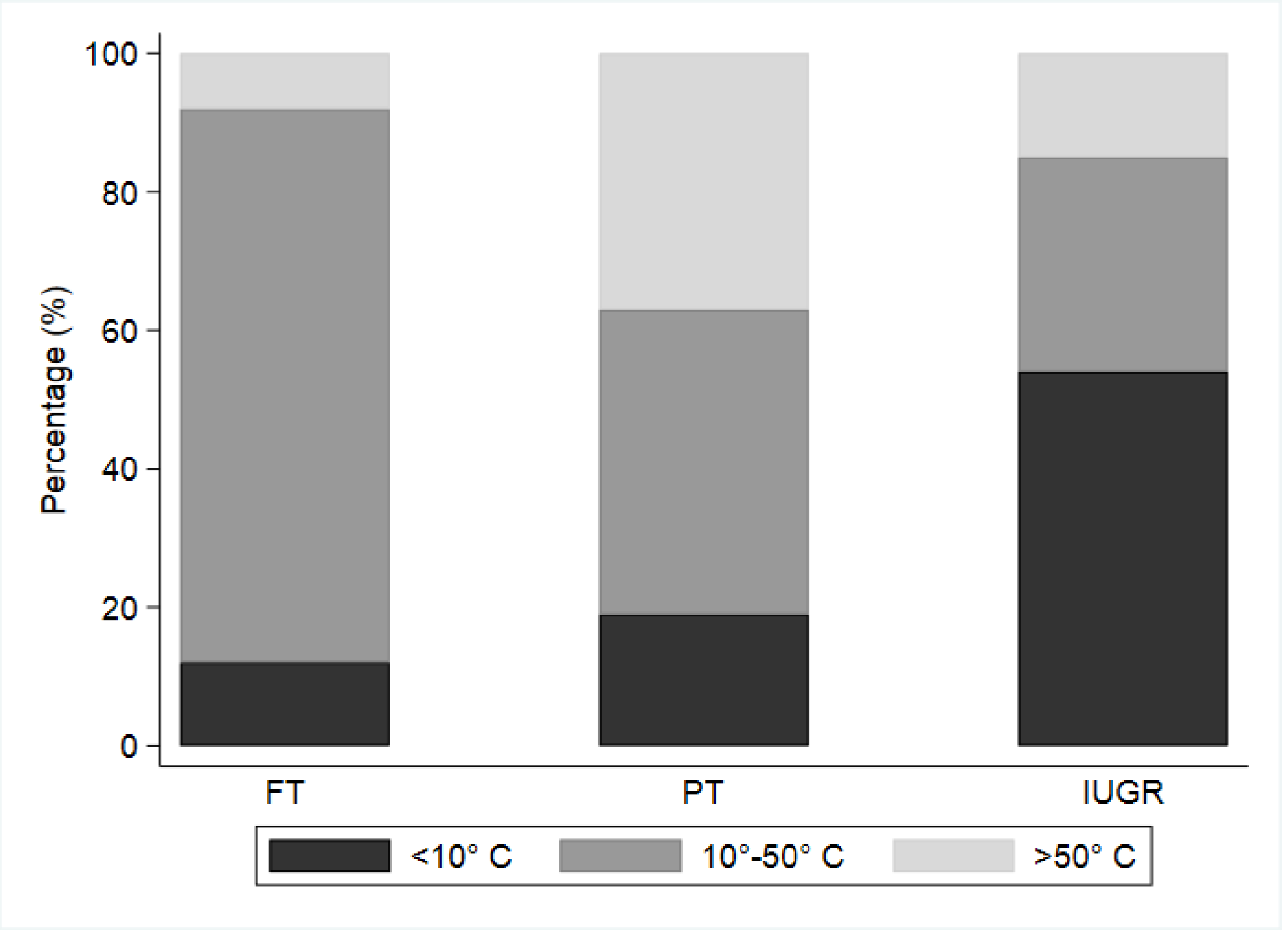

Figure S1. Distribution of weight percentiles across types of neonate through 12 months of age (Fisher's test p=0.016).
